# Longitudinal Cognitive Assessment in Low-Risk Very Preterm Infants

**DOI:** 10.3390/medicina58010133

**Published:** 2022-01-16

**Authors:** Domenico M. Romeo, Martina Ricci, Federica Mirra, Ilaria Venezia, Maria Mallardi, Elisa Pede, Eugenio Mercuri

**Affiliations:** 1 Pediatric Neurology Unit, Fondazione Policlinico Universitario A. Gemelli, IRCCS, 00168 Rome, Italy; riccimartina.rm@gmail.com (M.R.); fedemirra20@gmail.com (F.M.); venezia.ilaria@gmail.com (I.V.); maria.mallardi@guest.policlinicogemelli.it (M.M.); elisa.pede@hotmail.it (E.P.); eumercuri@gmail.com (E.M.); 2 Pediatric Neurology Unit, Universita Cattolica del Sacro Cuore, 00168 Rome, Italy

**Keywords:** low risk, preterm, cognitive outcome

## Abstract

*Background and Objectives*: Preterm infants are at higher risk of neurodevelopmental impairment both at preschool and school ages, even in the absence of major neurological deficits. The early identification of children at risk is essential for early intervention with rehabilitation to optimize potential outcomes during school years. The aim of our study is to assess cognitive outcomes at preschool age in a cohort of low-risk very preterm infants, previously studied at 12 and 24 months using the Griffiths scales. *Materials and Methods*: Sixty-six low-risk very preterm infants born at a gestational age of <32 weeks were assessed at 12 and 24 months corrected age using the Griffiths Mental Development Scales (second edition) and at preschool age with the Wechsler Preschool and Primary Scales of Intelligence (third edition) (WPPSI-III). *Results*: At 12 and 24 months and at preschool age, low-risk very preterm infants showed scores within normal ranges with similar scores in males and females. A statistically significant correlation was observed in the general developmental quotient between 12 and 24 months; a further significant correlation was observed between the early cognitive assessments and those performed at preschool age, with a better correlation using the assessments at 24 months. *Conclusion:* The present study showed a favourable trajectory of cognitive development in low-risk very preterm infants, from 12 months to preschool age.

## 1. Introduction

Preterm infants, i.e., infants born at <37 weeks of gestational age, have recently become more frequent due to several risk factors, including maternal ones (alcohol, drugs, diabetes, nephropathy, etc.), pregnancy complications, intra-uterine grow retardation, multiple births and due to the improvement of management and neonatal care [1].

In addition to the short-term complications, such as respiratory problems, hypoglycaemia, jaundice, sepsis and intraventricular haemorrhage, preterm infants are at increased risk of long-term sequelae, and mainly neurodevelopmental, sensory and motor impairments. [2,3]. The risk of morbidity depends on the level of immaturity in terms of gestational age and birth weight [4,5].

Even in preterm infants without major neurological deficits, minor impairments, such as mild motor problems, low intelligence quotient (IQ), learning disorder, linguistic delay and difficulties in many neuropsychological functions, occur in about 40–70%, both at preschool and school ages [4,6].

Psychomotor assessments from infant age to preschool and school age are relevant to the clinical evaluation of neurodevelopment at different ages.

In fact, the early identification of infants at risk of neurodevelopmental impairment is essential for early rehabilitation treatment to optimize potential outcomes during school years [7]. In early infancy, neurodevelopmental assessments are usually performed using the so-called baby tests, such as Griffiths or Bayley, though these are considered to be poor predictors of later IQ, and should be used as general indicators of development only and not as immutable information for long-term predictions [5]. Furthermore, because of the cerebral maturation that occurs in the first two years of life, development progresses rapidly in these years; therefore, it is essential to make serial assessments to correctly identify those infants with a real developmental delay. A more in-depth assessment of cognitive function at preschool or school age should be performed. At this age, the Wechsler scales represent the most widely used assessment instruments for determining a child’s intellectual abilities [7].

Although the cognitive development of very preterm infants has been widely studied both in infancy and at school age [5,6,7,8], only a few studies have reported information on longitudinal assessments [9,10], especially in low-risk preterm infants.

The aims of our study are to assess cognitive outcomes at preschool age in a cohort of low-risk very preterm infants, previously studied at 12 and 24 months using the Griffiths scales, and to evaluate the temporal stability of these assessments.

## 2. Materials and Methods

Infants were recruited from the Neonatal Intensive Care Unit at Gemelli Hospital, Rome, from 2012 to 2016. They were consecutively enrolled in a follow-up research program which included all infants born at a gestational age (GA) of < 32 weeks.

As part of this follow-up, all infants underwent a cranial ultrasound (cUS) during the first week from birth and at term age and were systematically assessed until preschool age.

Inclusion criteria were: (i) premature birth with GA at <32 weeks; (ii) normal or mildly abnormal cUS, such as grade 1 intraventricular haemorrhage (IVH) or transient periventricular echo density [11]; (iii) the absence of major visual impairment, and infants having retinopathy of prematurity (ROP) > stage 1; (iv) the ability to partake in longitudinal cognitive assessments at 12 and 24 months and at preschool age.

We excluded infants with major congenital malformations, genetic chromosomal abnormalities, metabolic disorders, congenital infections or any sign of encephalopathy.

Parental permission was obtained in all the cases. The ethical committee of our institution approved the study.

### 2.1. Neurodevelopmental Assessment at 12 and 24 Months

Infants were assessed at 12 and 24 months corrected age using the Griffiths Mental Development Scales (second edition) [12]. This is a standardised assessment for infants, consisting of five subscales, ‘Locomotor’ (A), ‘Personal-social’ (B), ‘Language production and comprehension’ (C), ‘Eye-hand coordination’ (D) and ‘Performance’ (E).

Developmental outcome was considered to be normal when the general developmental quotient (GDQ) was 85 or above, borderline if the GDQ was between 84 and 70 and delayed if the GDQ was less than 70.

### 2.2. Cognitive Function at Preschool Age

The Wechsler Preschool and Primary Scales of Intelligence (third edition) (WPPSI-III) was used to assess preschool cognitive function (in children between 4 years and 7 years 3 months) [13].

It provides three intelligence quotients: a performance IQ (PIQ), verbal IQ (VIQ) and full-scale IQ (FSIQ). 

All three IQ scores have a mean of 100 and a standard deviation of 15. Therefore, the intelligence quotient was considered to be normal when the FSIQ was 85 or above, borderline if the GDQ was between 85 and 70 and delayed if the GDQ was less than 70.

### 2.3. Statistical Analysis

Results were reported as mean ± standard deviation (SD). The correlation between the GDQ scores for the Griffiths at 12 and 24 months and the FSIQ scores obtained using the WPPSI-III were explored using the Pearson correlation test. Sensitivity and specificity were calculated to assess the predictive value of the GDQ scores at 12 and 18 months for the FSIQ scores obtained with the WPPSI-III test.

A comparison between genders for GA, birthweight, GDQ at 12 and 24 months and its subscales, VIQ, PIQ and FSIQ scores was assessed using the non-parametric test of Mann–Whitney U. The level of significance was set at *p* < 0.05.

## 3. Results

During the study period, a total of sixty-six (25 male, 41 female) low-risk preterm infants (GA: 29.3 ± 1.6 weeks; birthweight: 1132.2 ± 305.2 g) fulfilled the inclusion criteria and were included in the present study. 

Mildly abnormal US scans were observed in 24 infants (13 males, 11 females), 21 with transient periventricular echo densities and three with grade 1 IVH. No gender differences were observed for GA, birthweight or cUS scan findings using the non-parametric test of Mann–Whitney U.

All the infants performed three longitudinal neurodevelopmental/cognitive assessments. No gender or gestational age differences were found both for the Griffiths and WPPSI-III scores using the non-parametric test of Mann–Whitney U.

### 3.1. Neurodevelopmental Assessment at 12 and 24 Months 

Infants were assessed at 12 (mean 12.3 ± 1.3 months) and 24 months (mean 25.7 ± 3.5 months) CA. 

The mean general developmental quotient (GDQ) at 12 months CA was 111.6 ± 10 and at 24 months CA was 110.6 ± 15.5. 

The GDQ was ≥ 85 in all the infants but one at 12 months (GDQ = 79) and four at 24 months (three infants with GDQ = 84 and one GDQ = 83). No one reported a DQ < 70. Males and females reported similar scores (*p* > 0.05) on the Griffiths scales. No significant statistical difference (*p* > 0.05) was observed between the scores performed at 12 and 24 months. 

Details of the scores are described in Table 1 and in Table 2. 

A statistically significant correlation between GDQ at 12 and 24 months was observed (r = 0.47; *p* < 0.001). A lower but still significant correlation was also found among all the subscales (Table 3).

### 3.2. Cognitive Function at Preschool Age

All the sixty-six patients were re-assessed at preschool age using the WPPSI-III at a mean age of 57 ± 8 months.

The mean full-scale intelligence quotient (FSIQ) was 100.3 ± 15. The majority of children scored in the normal range, but seven children reported an FSIQ < 85, but none had an FSIQ < 70. Two infants reported a VIQ < 85 and one < 70; eight infants reported a PIQ < 85, and none had <70. Males and females reported similar scores (*p* > 0.05) on the WPPSI scale. Details of the scores are described in Table 4. 

### 3.3. Neurodevelopmental Scores at 12 and 24 Months and IQs at Preschool Age

Both GDQ scores at 12 and 24 months showed a statistically significant correlation with FSIQ (*p* < 0.05 for GDQ at 12 months, *p* < 0.001 for GDQ at 24 months) and with VIQ (*p* < 0.01 for GDQ at 12 months, *p* < 0.001 for GDQ at 24 months). GDQ scores at 12 months were not statistically significantly correlated with PIQ (*p* > 0.05), whereas scores at 24 months reported a statistically significant correlation with PIQ (*p* < 0.05). The predictive power was higher using the assessments at 24 months. 

**Figure 1:**
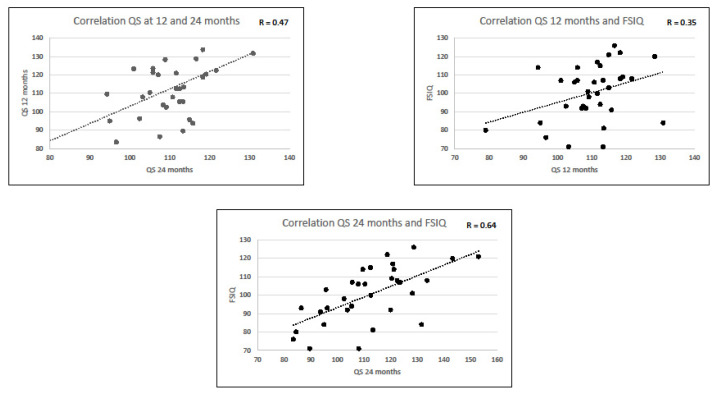
Correlation between Griffiths and the WPPSI-III scores.

The correlation coefficient between neurodevelopmental scores at 12 and 24 months and WPPSI scores are reported in Table 5 and Figure 1. Table 5 also reports the sensitivity and specificity of a GDQ score > 85 at 12 and 24 months for a normal IQ. 

## 4. Discussion 

Cognitive functions in preterm infants should be taken early to detect any developmental delay or differing patterns of development so that intervention programs may be provided. Although evidence exists that preterm infants may show cognitive impairments in different domains when compared to full-term ones, these are usually reported at lower gestational ages and in those preterm infants with major brain lesions [14,15,16]. However, low-risk preterm infants could have a high dropout in the follow-up, as they have no major complications (such as retinopathy or brain abnormalities), and little information is reported in literature on longitudinal cognitive data or on the stability of the assessments between infancy and preschool ages. 

Therefore, in this study, we described the longitudinal cognitive development of a population of low-risk very preterm infants at 12 months and 24 months CA and reassessed them at preschool age to evaluate the temporal stability of the assessments. 

Various standardized psychometric tests are available in infancy and toddlers during the first 2–3 years of age, describing a comprehensive evaluation of a child’s skills, including cognition, communication, motor skills, daily living skills and social and behavioural skills [7,17]. The Griffiths scales assessment, used in the present study, is considered to be one of the most used infant developmental tests in Western countries, especially in the follow-up of at-risk infants [17]. However, these scales are generally poorly predictive for later performance [7,17], and should be used only as general indicators of development. It is not until age 4–5 years that scores on standardized tests begin to show some stability [7]. Despite a lack of predictive validity, testing young children is helpful for detecting specific age-related abilities, determining the need for referral for further evaluation or treatment and monitoring early intervention and therapy [7,17].

On the other hand, the WPPSI-III is a cognitive or intelligence test for young children (from 2 years, 6 months through to 7 years, 3 months) with scores provided using an intelligence quotient (IQ); it emphasizes problem-solving and communication skills, rather than motor play and behavioural issues, with more predictive value for future intelligence [7]. 

Many studies in the literature have used Griffiths scales to assess the psychomotor development of preterm infants at early ages [8,18,19,20,21,22], but there are few data analysing the longitudinal development of the cognitive functions at 12 and 24 months and or at preschool age in those at low risk [9,10]. 

Our results show that more than 90% of low-risk very preterm infants reach scores within normal range from 12 months (using the Griffiths scales) to preschool age (using the WPPSI), and no one had scores <70. These data reinforce the idea that healthy condition is an important factor which prevents cognitive delay [14], with a general stability of cognitive performance shown between these two tools. Although low-risk preterm infants as a group have a more unfavourable outcome than term infants with lower cognitive scores [14,15,16], this is due to a small subgroup of low-risk preterm infants with moderate neurological abnormalities and/or poorer neuropsychological outcomes at age 5 years [23]. The remaining low-risk preterm infants show no neurological and/or neuropsychological disabilities compared with the term infants with similar cognitive scores at school age [14,23]. Our study confirms these data from the literature, showing that when excluding risk factors, such as neurological impairments, ROP or brain abnormalities, low-risk very preterm infants show neurodevelopmental and cognitive scores similar to the normative ones. 

In the present study, we observed a strong and significant correlation between global and subscale DQ scores at 12 and 24 months CA. Our results are in agreement with the study conducted by Sansavini et al. [10], in which 78 low-risk preterm infants were assessed using the Griffiths scale at 6–12–18–24 months CA; in this study, the authors found, similarly to our experience, a significant correlation between both psychomotor development at 12 months and outcome at two years, while there was no correlation between psychomotor development at 6 months and outcome at two years. 

In the present study, we were also able to correlate the scores obtained at 12 and 24 months CA with those obtained using the WPPSI-III at preschool age to relate the results of the infant test to a more in-depth assessment of cognitive competence. Our results showed a statistically significant correlation between GDQ at 12 and 24 months and both the FSIQ and the other intelligence quotients of the WPPSI, with the strength increasing over time. The only exception was with regards to the PIQ, for which there was not a statistically significant correlation with GDQ at 12 months.

Although the scores obtained at 12 months showed a good correlation with those obtained at preschool age using the WPPSI, the assessments at 24 months showed a higher predictive power, as previously reported [24,25,26,27]. Furthermore, the scores at 24 moths reported better sensitivity and specificity. This result is consistent with those of O’Connor et al. [24], who also evaluated the ability of early serial developmental assessments (Griffiths scale at 6–12–24 months) to predict preschool outcome (WPPSI-III at 5 years) in a population of infants with hypoxic-ischaemic encephalopathy, without therapeutic hypothermia. The authors revealed the presence of a statistically significant correlation between GDQ at all ages and both FSIQ and the single subscale quotients, with the correlation increasing with increasing age. Furthermore, and consistent with our findings, Barnett et al. [25], in a previous neonatal encephalopathy cohort, also demonstrated higher prediction at 24 months with the GDQ for the WPPSI total quotient than the GDQ at 12 months.

In our cohort, we observed an increase in borderline scores with the increase in the age; namely, at 12 and 24 months, one and four infants, respectively, reported a score between 70 and 84, while at preschool age, seven children had scores within this range. This could be explained by the fact that with increasing age, more difficulties could be observed, especially in the language areas. For this reason, it seems to be important to conduct serial early assessments in the first two years of life. [9,22]. Wong et al. [9] suggest that early developmental assessments have poor sensitivity but good specificity and negative predictive value for school-age cognitive deficits; these early assessments are generally accurate at predicting the absence of school-age cognitive deficits, but the identification and prediction of children who would have cognitive difficulties are weak.

In the present study we also explored the role of gender. No gender differences were found both for the Griffiths and WPPSI-III scores. This result is not in agreement with previous studies [28,29] that reported better scores in females. This could be explained by the female/male ratio in our sample (F:M = 1.6:1) and by the selection of our study, due to the inclusion criteria, to create a more homogeneous group without specific neurological damage. Gender differences were not observed in early and late cognitive assessments, indicating that gender is not a predictive factor of cognitive development in the absence of major neurological impairments.

A limitation of the study is that the assessment tools used in the present study, although validated and contemporary at the time of the assessments, have been superseded by newer editions. However, although psychometric property differences exist, all the assessment tools provide comparative information of an individual’s development in reference to age-appropriate normative data on the same scale [9].

Another limitation of our study is the small sample size due to the retrospective monocentric design of the present study. 

This study, although performed with a small number of infants, suggests a stability of cognitive profiles within the normal range from 1 to 5 years in a population of low-risk very preterm infants.

Further studies are needed to confirm our data, even at older ages, in larger cohorts of preterm infants at both low and high risk. 

## Figures and Tables

**Table 1:** Griffiths scales scores at 12 months.

	SQ-A (Mean ± SD)	SQ-B (Mean± SD)	SQ-C (Mean ± SD)	SQ-D (Mean ± SD)	SQ-E (Mean ± SD)	GDQ (Mean ± SD)
Total	106 ± 15	115 ± 13.4	112,3 ± 16	110 ± 12.1	115 ± 16.7	111.5 ± 10
Female	105.7± 14.3	116 ± 13.8	112.1 ± 15.8	109.4 ± 12	114.5 ± 11.8	111.5 ± 9.7
Male	106.3 ± 16.6	112.7 ± 12.7	112.6 ± 16.3	111 ± 12.5	115.3 ± 22.8	111.6 ± 10.7

SQ: subscale quotient; SD: standard deviation; GDQ: general developmental quotient.

**Table 2:** Griffiths scales scores at 24 months.

	SQ-A (Mean ± SD)	SQ-B (Mean ± SD)	SQ-C (Mean ± SD)	SQ-D (Mean ± SD)	SQ-E (Mean ± SD)	GDQ (Mean ± SD)
Total	107.9 ± 20	120.6 ± 21.5	109.7 ±22.5	103.2 ± 13.2	111.3 ± 20.4	110.5 ± 15.5
Female	106.9 ± 21.3	120.3 ± 21.5	109.4 ±24.2	102 ± 13.4	110.2 ± 19.9	109.7 ± 16
Male	109.5 ±17.8	121.1 ± 22	110.1 ± 20	105.3 ± 13	113.2 ±21.5	111.8 ± 15

SQ: subscale quotient; GDQ: general developmental quotient.

**Table 3:** Pearson correlation test between neurodevelopmental scores at 12 and 24 months.

SQ-A 12 m	SQ-A 24 m	SQ-B 12 m	SQ-B 24 m	SQ-C 12 m	SQ-C 24 m	SQ-D 12 m	SQ-D 24 m	SQ-E 12 m	SQ-E 24 m	GDQ 12 m	GDQ 24 m
r = 0.40		r = 0.32		r = 0.30		r = 0.28		r = 0.30		r = 0.47	
*p* < 0.001		*p* < 0.01		*p* < 0.05		*p* < 0.05		*p* < 0.05		*p* < 0.001	

**Table 4:** WPPSI-III scores.

	VIQ (Mean ± SD)	PIQ (Mean ± SD)	FSIQ (Mean ± SD)
Total	103.3 ± 15.1	98.9 ± 16.3	100.3 ± 15
Female	100.6 ± 14.3	99 ± 17.7	99.2 ± 16
Male	107.9 ± 16	98.7 ± 14.4	102.3 ± 13.4

VIQ: verbal intelligent quotient; PIQ: performance intelligent quotient; FSIQ: full-scale intelligence quotient.

**Table 5:** Pearson correlation test between GMDS at 12–24 months and WPPSI scores.

	GDQ 12 m	GDQ 24 m
Correlation coefficient		
FSIQ	0.35 (*p* < 0.05)	0.64 (*p* < 0.001)
VIQ	0.44 (*p* < 0.01)	0.60 (*p* < 0.001)
PIQ	0.05 (*p* > 0.4)	0.40 (*p* < 0.05)
Sensitivity/Specificity		
FSIQ	0.2/1	0.6/1
VIQ	0/0.98	0.6/0.98
PIQ	0/0.98	0.38/0.98

## Data Availability

Data available on request due to restrictions eg privacy or ethical.
